# ***In Memoriam***  Robert Ellis Shope  **1929–2004**

**DOI:** 10.3201/eid1004.040156

**Published:** 2004-04

**Authors:** Frederick A. Murphy, Charles H. Calisher, Robert B. Tesh, David H. Walker

**Affiliations:** * University of California – Davis, Davis, California, USA; † Colorado State University, Fort Collins, Colorado, USA; ‡ The University of Texas Medical Branch, Galveston, Texas, USA

**Figure:**
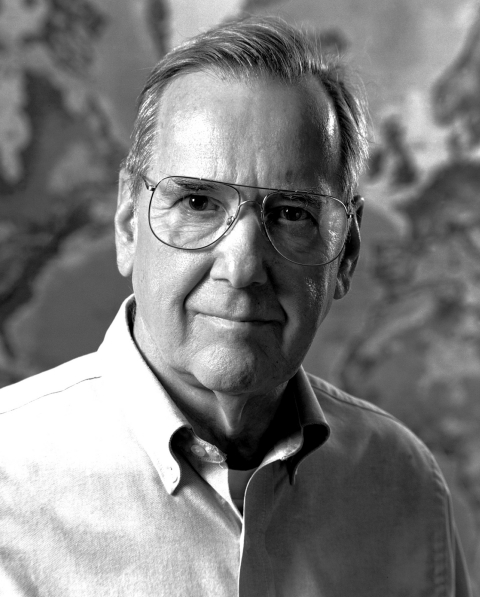
Robert Ellis Shope **1929–2004**

Robert Ellis Shope, one of the world’s most distinguished arbovirologists and a dear friend of many colleagues around the world, died of complications of idiopathic pulmonary fibrosis in Galveston, Texas, on January 19, 2004, at age 74. Bob is survived by his wife, Virginia; his daughters, Deborah Shope and Bonnie (Shope) Rice; his sons, Peter and Steve; his brothers, Thomas and Richard; his sister, Nancy (Shope) FitzGerrell; and six grandchildren.

It is difficult to describe Bob’s many contributions to virology, epidemiology, tropical medicine, infectious disease sciences, vector biology, and international public health because they are so numerous and varied. His lifelong contributions to our understanding of arthropod-borne viruses, hemorrhagic fever viruses, and the diseases these viruses cause are without equal. He discovered and characterized more previously unknown viruses than any other person in history. Working at various times in nearly every country where these viruses and diseases are important, he collaborated with virtually everyone who has worked in these fields in the past 50 years. Until his death, he remained an international leader in framing the global response to emerging and reemerging diseases and our national response to bioterrorism, while at the same time keeping his own laboratory productive—his research was funded continuously by the National Institutes of Health (NIH) for 26 years.

Arguably, Bob’s most important contribution was his co-chairing, along with Joshua Lederberg and Stanley Oaks, of the Institute of Medicine Committee on Emerging Microbial Threats to Health. The proceedings of this committee led to the publication in 1992 of Emerging Infections: Microbial Threats to Health in the United States (National Academy Press). This seminal publication, which outlined factors implicated in the emergence of infectious diseases and the programs and resources needed to cope with them, initiated much of the current worldwide interest in infectious diseases. He then spent endless days explaining the concepts underpinning the report in order to gain public and political support. His efforts were marked by great success, as evidenced by the revitalized state of the infectious disease sciences today.

Bob was born in Princeton, New Jersey, the son of Richard Shope, an internationally renowned virologist. He received BA and MD degrees from Cornell University and completed an internship in internal medicine at Grace-New Haven Hospital (Yale University School of Medicine). He then spent 3 years in the U.S. Army Medical Corps, where he was initially assigned to Camp Detrick (now the U.S. Army Medical Research Institute for Infectious Diseases) and later to the U.S. Army Medical Research Unit in Kuala Lumpur. The latter experience, involving studies on the etiology of fevers of unknown origin among British soldiers and the local Malaysian civilian population, had a profound effect on his subsequent research interests and career decisions. After starting a residency in internal medicine at Yale, he left to take a staff position with the Rockefeller Foundation’s International Virus Program in its laboratory in Belem, Brazil (now the Instituto Evandro Chagas). There he remained for 6 years, eventually serving as director of that institute. This was a time of great excitement and discovery, as many new viruses were being isolated and characterized. In 1965, Bob returned from Brazil to Yale, where most of the senior staff of the Rockefeller Foundation’s overseas virus program had relocated and were establishing the Yale Arbovirus Research Unit (YARU). Bob remained at Yale for 30 years, rising to the rank of professor and director of that research unit.

In 1995, Bob moved to the University of Texas Medical Branch in Galveston, where he held several appointments: professor (Department of Pathology, Department of Microbiology and Immunology, Department of Preventive Medicine and Community Health), associate director of the university’s Center for Biodefense (and John S. Dunn Distinguished Chair in Biodefense), member (Sealy Center for Environmental Health & Medicine, Sealy Center for Structural Biology, World Health Organization [WHO] Collaborating Center for Tropical Diseases).

At various times throughout his long career, Bob served as president and councilor, American Society of Tropical Medicine and Hygiene; chair and member, Advisory Council, James A. Baker Institute for Animal Health, Cornell University; member, WHO Expert Panel on Virus Diseases, U.S. Delegation to the U.S.–Japan Cooperative Medical Science Program, International Committee on Taxonomy of Viruses (ICTV), Armed Forces Epidemiology Board, Advisory Board (Fogarty International Center, National Institutes of Health), Institute of Medicine Committee on Improving Civilian Medical Response to Chemical and Biological Terrorism Incidents, Advisory Committee (American Museum of Natural History Infectious Disease Exhibition), Advisory Panel (National Research Council Program on Strategies to Protect the Health of Deployed U.S. Forces), and the National Research Council Committee on Climate, Ecosystems, Infectious Diseases, and Human Health.

Over the years, Bob earned many honors, including the Bailey K. Ashford Award from the American Society of Tropical Medicine and Hygiene, the Richard M. Taylor Award from the American Committee of Arthropod-Borne Viruses, and the Walter Reed Medal from the American Society of Tropical Medicine and Hygiene.

In November 2003, the University of Texas Medical Branch celebrated the completion of a biosafety level 4 laboratory, the first in the United States at an academic institution. In Bob’s honor, the laboratory was named “The Robert E. Shope, M.D. Laboratory.” Also in his honor, the university established the Robert E. Shope, M.D. Memorial Fellowship in emerging viral diseases research.

## Professional Odyssey

Bob’s first assignment with the Rockefeller Foundation in New York in 1954 was to study the etiology of “epidemic polyarthritis,” a disease characterized by fever, arthralgia, and rash that occurred mainly during the summer in coastal regions of Australia. In a classic retrospective serologic investigation, he and S.G. Anderson demonstrated that the disease was caused by an alphavirus. Ten years later, Australian scientists, led by Ralph Doherty and Ian Marshall, isolated Ross River virus and confirmed its association with epidemic polyarthritis.

In the early 1960s, during Bob’s tenure at what is now the Instituto Evandro Chagas, he isolated and characterized more than 50 tropical arboviruses, most of which were new to science. Several, such as group C and Guama viruses, caused human disease. Not only did he play a role in isolating and characterizing these agents and their diseases, but his team was instrumental in studying and understanding the role of their forest reservoirs. He refined the capture-mark-release-recapture technology for rodents, marsupials, and birds, and applied these methods to the Amazon fauna. His team also correlated vertebrate reservoir ecology with that of vector mosquitoes. He identified Oropouche virus when the first epidemic struck the city of Belem. This disease has since emerged as a major scourge, not only in the Brazilian Amazon, but also in cities in Panama and Peru.

During his early tenure at the Yale Arbovirus Research Unit, Bob was involved in a collaborative project with the Smithsonian Institution and discovered several new arboviruses transported by birds migrating through the Nile Delta. Then, in 1969, Lassa fever and yellow fever emerged in Nigeria and occupied his research for several years.

Bob was first to show that rabies virus was related to other viruses (the “rabies-related viruses”); in 1970, with Fred Murphy and others, he characterized Mokola, Lagos bat, and Duvenhage viruses, the founding members (along with rabies virus) of the genus *Lyssavirus*, family *Rhabdoviridae*. In 1971, Bob, Fred Murphy, and Ernie Borden characterized many bluetongue-like viruses and established the genus *Orbivirus*, family *Reoviridae*. In 1971, Bob coauthored an obscure paper in the Indian Journal of Medical Research describing Thottapalayam virus, a presumed arbovirus isolated from a shrew in India. Thottapalayam virus was subsequently shown to be a hantavirus, the first hantavirus to be isolated (Hantaan virus, the type virus of the genus *Hantavirus,* family *Bunyaviridae*, was not isolated until 1976).

In 1977, Bob identified a virus isolated from a mixed pool of midges (culicoids) collected near Darwin, Australia, and sent to him by Toby St. George. Bob identified it as bluetongue 20 virus, the first bluetongue virus recognized in Australia. This discovery caused economic turmoil in the Australian livestock industry, but it was seminal in initiating an intensive research program in Australia. That same year, Bob and Jim Meegan identified Rift Valley fever virus as the cause of a “virgin soil” epidemic in Egypt that affected 200,000 people, with more than 600 human deaths, and hundreds of thousands of sheep and cattle. Also noteworthy is that in 1977, Bob co-authored the first description of Lyme disease in the United States. This work was done with Allan Steere, then a young rheumatologist at Yale. Lyme disease was subsequently recognized as the most important tick-borne disease in North America.

Throughout the most recent 20 years, Bob and his colleagues, especially Bob Tesh, continued to discover new, important viruses. These included Sabiá, the cause of Brazilian hemorrhagic fever, and Guanarito, the cause of Venezuelan hemorrhagic fever. Bob also worked to develop an attenuated-live virus vaccine for dengue and chaired the WHO advisory committee on dengue vaccine development.

After moving to the University of Texas Medical Branch in 1995, Bob embarked on a new phase of his career; he and his colleagues initiated a new program on bioterrorism countermeasures, centered on novel antiviral drugs against alphavirus, flavivirus, and arenavirus diseases. At the same time, along with Bob Tesh, Bob brought the World Reference Center for Emerging Viruses and Arboviruses to the university—this is one of the most valuable virus collections in the world, comprising >4,000 arthropod-borne virus strains and >1,000 other virus strains.

## Scientific Legacy

In addition to the many scientific contributions already mentioned, Bob wrote and edited more than 170 refereed papers and more than 90 books, chapters, and monographs. Most notable of his editorial activities was his contribution to several editions of the definitive reference book in virology, Fields Virology (Bernard N. Fields, editor; David Knipe, Robert Chanock, Joseph Melnick, Bernard Roizman, Robert Shope, Martin Hirsch, Thomas Monath, Peter Howley, and Stephen Straus, co-editors; Lippincott Williams & Wilkins).

Bob’s oral contributions are legion. Over the years, he presented many hundreds of seminars, lectures, and workshops throughout the world, but he never kept track of them. One can only guess at the numbers, but it is easy to recall their overall impact in many disciplines of medical science.

## Legacy as a National and International Advisor and Consultant

Bob served the national and international interests of science in many ways—he was an outstanding spokesman, always willing to take extra time and effort to “educate” politicians and science leaders. He did this at an ever-higher level of impact: in 1997 Bob was invited to the White House along with six other scientists, three of them Nobel laureates, to brief President Bill Clinton, Vice President Al Gore, and others on the perils of global warming and its importance in the spread of infectious agents, such as those causing dengue and dengue hemorrhagic fever, malaria, and many other arthropod-borne diseases.

Bob served as consultant and advisor to many national and international organizations. Throughout his career he was a frequent advisor to the NIH, Centers for Disease Control and Prevention, Department of Defense, U.S. Department of Agriculture, U.S. Agency for International Development, the Institute of Medicine, and the American Type Culture Collection. He was also a frequent consultant to WHO and the Pan American Health Organization, as well as to foreign governments.

## Legacy as a Teacher and Mentor

Bob was an outstanding, devoted, and beloved teacher. He cared deeply about the next generation of professionals entering the various fields of the clinical infectious disease sciences, virology, vector biology, epidemiology, and public health. His honesty, humility, enthusiasm, and caring manner endeared him to his students. He treated everyone with respect and was never too busy to meet with a student or to offer guidance. But there was more; there was a kind of magic in his “touch” as teacher and mentor. For example, Bob participated for many years in the Cornell Summer Leadership Program, run lovingly by Doug McGregor. At the end of a day spent in workshops, the students would gather in a lounge for a beer and informal discussion with the faculty. Within minutes, other guest faculty members were forgotten as the students zeroed in on Bob—they instinctively knew that he was the utmost rolemodel, an inspiration in deciding career directions.

## Legacy as a Colleague and Friend

Over the years, Bob worked with many prominent arbovirologists and vector biologists, including, in his tenure at Yale, Tommy Aitken, John Anderson, Sonia Buckley, Jordi Casals, Delphine Clarke, Wilbur Downs, Max Theiler, Loring Whitman, Jack Woodall, Barry Beaty, Rebecca Rico-Hesse, Dennis Knudson, Barry Miller, Thomas Schwann, Thomas Scott, Mark Wilson, and others. During his tenure at the University of Texas Medical Branch, he worked closely with Dave Walker, Bob Tesh, C.J. Peters, Stan Lemon, Scott Weaver, Alan Barrett, Judith Aronson, Chuck Fulhorst, Norb Herzog, Steve Higgs, Peter Mason, Doug Watts, Larry Stanberry, and others. But, such lists are folly—Bob’s true legacy as a colleague and friend would require a list of hundreds of names, from across the years and around the world. It would be wonderful to append such a list here, but it would be suspect in its likely omissions.

The essence of Bob’s legacy as a colleague and friend is the most difficult matter to capture here. After his death, condolences to his colleagues and family contained oft-repeated themes:

“He was the nicest, humblest, most self-effacing, and obliging person I have ever known…”

“He was unfailingly kind and generous…”

“He was always collegial with collaborators and endlessly patient with students; it is not surprising that he earned both the respect and love of both…”

“He consistently exemplified and fostered an attitude of service…”

“He always made time for you, whether you were a student or the minister of health…”

“He was always reassuring, upbeat and helpful…”

“He was an exceptionally generous person, and that tended to rub off on others…”

“His enthusiasm and love for his chosen field were as infectious as the viruses he studied…”

“Those of us who knew him and worked with him were very fortunate…”

“He was a catalyst of honesty, professionalism, and mutual respect….”

“We all benefited from his example…”

“He was the best listener in the world…”

“He always acted in the best interests of others, truly practicing the golden rule of treating others as he would wish to be treated…”

“We were blessed to have had him as a colleague...”

Running through these messages is the understanding that the field of arbovirology and related sciences has been incredibly satisfying to the involved community because of the warmth of personal relationships and the high ethical standard shared by one and all. In these messages, it is clear that everyone knew that Bob was the “keeper of the flame,” the exemplar, the strong positive influence on everyone in this community. This was not a passive matter—in the most subtle way Bob vaccinated others with his high ethical standard—magic, again!

The messages received in Galveston upon Bob’s death capture another theme, one based in each person’s memories of his or her earliest days in the world of medical research and public service. It seems that many, many people have a clear memory of their first experience with Bob. Two messages, anonymously paraphrased here, are exemplary. First, “…In the 1970s I went to YARU with a few viruses that had been brought back from Kenya and couldn’t be identified. I was new to the field and had little clue how to go about determining what these viruses were. But I had landed in the World Reference Center! Bob took me under his wing. He showed me the reference collection, inventoried on a huge Rolodex. We talked about the possibilities based on the source of the unknown viruses. Bob stayed with me until 10 p.m. developing a testing plan, and then drove me to my hotel. My head was spinning. YARU was a busy place and there were many other much more important visitors, but Bob worked closely with me over the week until we had identified all of the viruses. The result was one of my first publications in arbovirology…”

Second, “…I well remember as a young nobody from the outback of Africa arriving to consult with Bob at YARU in 1975. I wanted some reference arbovirus reagents to take home with me, and having just met me, Bob worked with me late into the evening, freeze-drying viruses and antigens so that I could leave with them on the following day…”

Does all this capture Bob Shope, the man, the friend that we have lost? If not, then words cannot serve what memories can. To say that Bob will be missed is the greatest of understatements—he will never be forgotten. He leaves to us a legacy of enthusiastic commitment to his science and an equal commitment to the application of that science for the benefit of the people of the world. As well, he leaves to us, by the example of his life, a definition of our sense of community and the keys to its nurturing.

